# Supplementary material for: Phase-driven charge manipulation in Hybrid Single-Electron Transistor
Source: Sci Rep. 2017 Oct 18;7:13492. doi: 10.1038/s41598-017-13894-z (PMC5647419; doi:10.1038/s41598-017-13894-z)
Supplement: Supplementary file 1 — Supplementary Information [file 41598_2017_13894_MOESM1_ESM.pdf]

Supplementary Information

Phase-driven charge manipulation in Hybrid Single-Electron Transistor

Emanuele Enrico, Elia Strambini, Francesco Giazotto

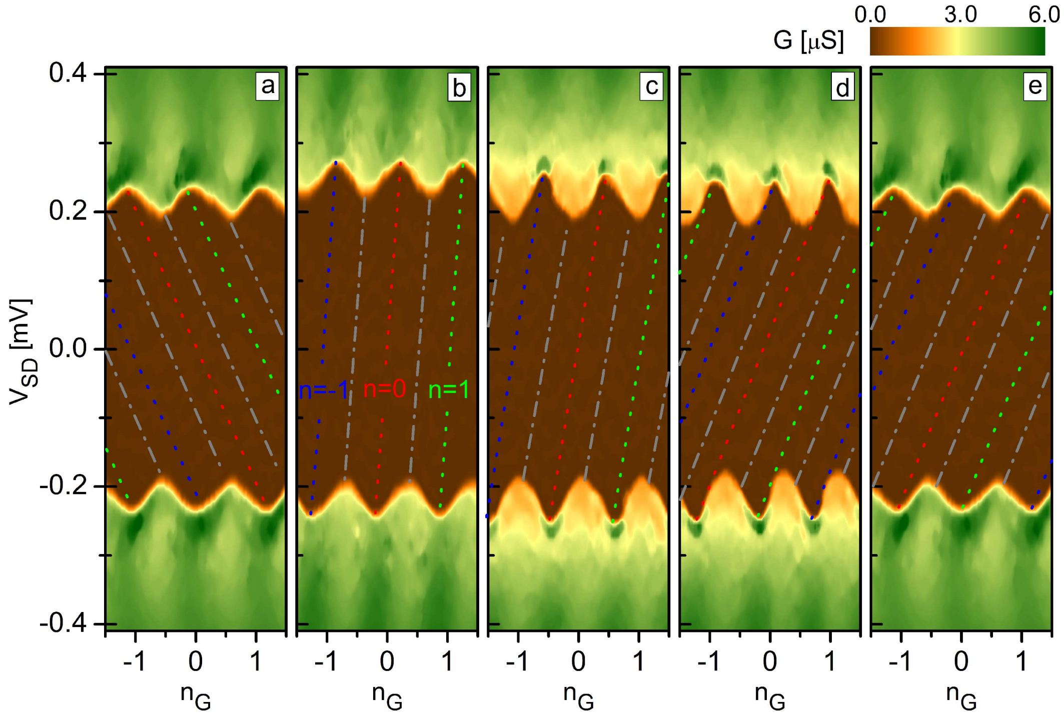

Supplementary Figure S1. Stability diagrams under turnstile behavior

a-e, stability diagrams showing the measured differential conductance ($\boldsymbol{G} = \partial \boldsymbol{I}_{SD}/\partial \boldsymbol{V}_{SD}$). Gray dash-dotted lines correspond to blockaded regions having fixed island charge configuration ($\boldsymbol{n}$ in **b**). RGB lines (corresponding to energy configurations in Fig.3 **a-c** of the manuscript) are guidelines for different charge states (expanded diamonds). **a-e** have been measured for $\boldsymbol{\Phi_B} = \mathbf{0.359}\,\boldsymbol{\Phi_0}$, $\boldsymbol{\Phi_B} = \mathbf{0.393}\,\boldsymbol{\Phi_0}$, $\boldsymbol{\Phi_B} = \mathbf{0.428}\,\boldsymbol{\Phi_0}$, $\boldsymbol{\Phi_B} = \mathbf{0.463}\,\boldsymbol{\Phi_0}$ and $\boldsymbol{\Phi_B} = \mathbf{0.5}\,\boldsymbol{\Phi_0}$ respectively. In **b** the superconducting gaps of both source and drain electrodes are almost fully open while in **a** (and **e**) they are in the antiphase case being the first open and the second closed (vice versa in **e**). All measurements were taken at 21 mK of bath temperature.
